# IRF4/FGF23 in gingival crevicular fluid: Diagnostic biomarkers for Dentin Hypersensitivity

**DOI:** 10.5937/jomb0-60969

**Published:** 2026-03-17

**Authors:** Song Can, Jia Yubao, Liu Yaxuan, Zhang Yujie, Yu Jing, Xian Mengmeng

**Affiliations:** 1 The Second Hospital of Shijiazhuang, Department of Stomatology, Shijiazhuang, Hebei, China

**Keywords:** IRF4, FGF23, Dentin Hypersensitivity, inflammatory cytokines, T lymphocyte subsets, IRF4, FGF23, preosetljivost dentina, inflamatorni citokini, podskupovi T limfocita

## Abstract

**Background:**

The aim of this study is to investigate the diagnostic value of interferon regulatory factor 4 (IRF4) and fibroblast growth factor 23 (FGF23) in gingival crevicular fluid (GCF) and serum for dentin hypersensitivity (DH), and to analyze the relationship between IRF4, FGF23 and inflammatory factors and T lymphocyte subsets.

**Methods:**

24 DH patients receiving orthodontic treatment at our institution between 2022 and early 2025 were enrolled as the study cohort, along with 124 healthy controls matched for age and sex. GCF and serum samples were obtained 48 hours post-desensitization therapy to quantify IRF4 and FGF23 levels (via enzyme-linked immunosorbent assay), along with serum inflammatory markers (interleukin [IL]-1β, IL-6, high-sensitivity C-reactive protein [hs-CRP]) and T-cell subpopulations (CD3+, CD4+, CD8+). Statistical analyses included Pearson's correlation to examine relationships between IRF4/FGF23 and clinical parameters, with receiver operating characteristic (ROC) curve analysis determining diagnostic accuracy.

## Abstract (sr)

**Uvod:**

Cilj ove studije je ispitivanje dijagnostičke vrednosti regulatornog faktora interferona 4 (IRF4) i faktora rasta fibroblasta 23 (FGF23) u tečnosti gingivalnog sulta (GCF) i serumu za preosetljivost dentina (DH), kao i analiza odnosa između IRF4, FGF23 i inflamatornih faktora i podskupova T limfocita.

**Metode:**

U studiju je uključeno 24 pacijenta sa DH koji su primali ortodontski tretman u našoj ustanovi između 2022. i početka 2025. godine, zajedno sa 124 zdrave kontrolne osobe uparene po starosti i polu. Uzorci GCF i seruma su dobijeni 48 sati nakon terapije desenzibilizacije kako bi se kvantifikovali nivoi IRF4 i FGF23 (putem imunosorbentnog testa povezanog sa enzimom), zajedno sa serumskim inflamatornim markerima (interleukin [IL]-1β, IL-6, C-reaktivni protein visoke osetljivosti [hs-CRP]) i subpopulacijama T-ćelija (CD3+, CD4+, CD8+). Statističke analize su uključivale Pirsonovu korelaciju za ispitivanje veza između IRF4/ FGF23 i kliničkih parametara, pri čemu je analiza ROC krive određivala dijagnostičku tačnost.

## Introduction

Dentin hypersensitivity (DH) is a prevalent oral condition marked by brief, intense pain in exposed dentin when triggered by external stimuli such as temperature changes, acidic or sweet foods, or mechanical contact [1]. Its widespread occurrence negatively impacts patients’ daily lives and oral hygiene practices. According to the hydrodynamic theory, DH-induced pain arises from the opening of dentinal tubules and the movement of fluid within them [2]. However, clinical observations reveal considerable variability in sensitivity intensities and durations among patients with comparable levels of dentin exposure [3]. These findings highlight that, beyond structural tubule patency, localized biological microenvironment changes (e.g., inflammatory and metabolic disturbances) could be key, though overlooked, drivers of DH initiation, progression, and persistence [4][5]. Clarifying these molecular mechanisms is therefore essential for advancing the understanding of DH pathology and improving diagnostic and therapeutic approaches.

Interferon regulatory factor 4 (IRF4), a critical transcription factor that modulates immune cell (e.g., lymphocytes and macrophages) development and activity, is pivotal in maintaining inflammatory balance and immune system stability [6]. It is worth noting that recent studies suggest that IRF4 is a pivotal gene and transcription factor in periodontitis [7]; however, its expression profile and functional relevance in DH, a distinct pain condition, remain unexplored. Fibroblast growth factor 23 (FGF23), primarily produced by osteocytes, serves as a central regulator of phosphate homeostasis and bone mineralization [8]. In dental research, increased FGF23 has been strongly linked to periodontal tissue degradation and alveolar bone loss [9]. Despite the structural parallels between dentin and alveolar bone as mineralized tissues [10], the potential involvement of FGF23 in modulating the microenvironment of exposed dentin and its subsequent effects on dentinal tubule permeability and nociceptive sensitivity represents a scientifically valuable area for investigation.

The local microenvironment of periodontal tissues surrounding exposed dentin is thought to involve immune activation (mediated by IRF4) and disruptions in bone/dentin metabolism (associated with FGF23). These alterations may influence the composition of gingival crevicular fluid (GCF), potentially contributing to DH onset and progression. However, systematic investigations into the expression patterns of IRF4 and FGF23 in DH and their relationship with clinical manifestations remain scarce. To address this gap in knowledge, the present study compared two diagnostic methods of DH, either by serum or by GCF measurement of IRF4 and FGF23 expression. The aim is to provide a new idea for the clinical diagnosis of DH in the future.

## Materials and methods

### Participants

This investigation enrolled individuals diagnosed with DH who were treated at the Stomatology Department of our medical institution between 2022 and early 2025. Sample size determination was performed utilizing G-Power v3.1.9.2 statistical software (effect size = 0.3 based on prior GCF biomarker studies [11], α = 0.05, power = 0.95), indicating a minimum requirement of 111 participants. Subsequent participant selection was conducted based on predefined eligibility and exclusion criteria. Eligibility criteria: The age ranged from 18 to 30 years, both sexes; Clinical confirmation of DH diagnosis [12]; Selected teeth exhibiting probing depth (PD) ≤ 3 mm with either no clinical attachment loss (CAL) or CAL ≤ 1 mm; Radiographic verification confirming absence of dental caries, fillings, fractures, pulp inflammation, or periapical pathology in target teeth. Exclusion criteria: Recent use (within 3 months) of desensitizing toothpaste or desensitization therapies; Consumption of pain relievers, anti-inflammatory medications, or immunosuppressive drugs within the preceding month; Women who were pregnant or breastfeeding; Presence of systemic conditions (including poorly controlled diabetes, autoimmune disorders, chronic renal disease, or bone metabolism abnormalities); Prolonged usage of medications influencing bone metabolism or immune response (e.g., bisphosphonates, corticosteroids); Target teeth demonstrating bruxism or significant wear due to malocclusion. The final study cohort comprised 124 DH patients (DH group). For comparative analysis, an equal number of age – and sex-matched healthy individuals (DH group: control group = 1: 1) were recruited as controls by propensity score matching (PSM) during the same time period (caliper value = 0.2), and these healthy individuals were undergoing routine health examination. Healthy controls: No dental plaque (PI<1), probing depth ≤3 mm, no periodontitis.

After PSM, the DH and control groups showed balanced baseline characteristics, with no significant disparities in age or gender distribution (P > 0.05). Additionally, factors such as history of oral diseases, smoking habits, and alcohol consumption were similarly distributed between the two groups (P > 0.05), ensuring their comparability (Table 1).

**Table 1:** Clinical data.

	Control group (n=124)	DH group (n=124)	Statistics	P
Age (years)	23.74±3.31	23.89±3.79	t=0.321	0.748
Gender			χ^2^=1.106	0.293
male	74 (59.68)	82 (66.13)		
female	50 (40.32)	42 (33.87)		
History of oral diseases			χ^2^=1.297	0.255
yes	30 (24.19)	38 (30.65)		
no	94 (75.81)	86 (69.35)		
Smoking			χ^2^=0.413	0.520
yes	22 (17.74)	26 (20.97)		
no	102 (82.26)	98 (79.03)		
Drinking alcohol			χ^2^=0.575	0.315
yes	15 (12.10)	18 (14.52)		
no	109 (87.90)	106 (85.48)		
Body Mass Index (kg/m^2^)	21.48±1.39	21.34±1.51	t=0.760	0.448

### Statement of ethics

The institutional ethics review board granted approval for this research, with written informed consent obtained from all participants prior to enrollment.

### Collection of test samples

Serum samples: 3 mL of venous blood was drawn from each individual. Serum was collected using a procoagulant tube and allowed to stand for 30min at room temperature before centrifugation (1505 × g for 15 min) to separate serum. All serum samples were immediately preserved at -80°C to ensure stability for future laboratory testing.

GCF samples: After gargle with normal saline, the target tooth was exposed (the most obvious lesion site was selected). The filter paper strip (Periopaper, Oraflow Inc.) was clamped with sterilized forceps and inserted slowly (1–2 mm) along the gingival sulcus floor and allowed to stand for 30 – 60 s (adsorption). The filter paper strip was gently removed with forceps and immediately placed into a frozen storage tube containing preservation solution (PBS buffer, pH 7.2-7.4, containing 0.05% sodium azide for antiseptic). The supernatant was obtained by incubation with RIPA buffer for 60 min (4°C) and centrifugation (1000 × g for 5 min) for subsequent assays.

### Laboratory analyses

GCF and serum IRF4 (Cusabio CSB-E13066h) and FGF23 (R&D Systems DFG230) concentrations were quantified using enzyme-linked immunosorbent assay (ELISA). Prior to analysis, frozen samples were gradually thawed at 4°C. They were then mixed with 300 μL of elution buffer (supplied with the kit) and shaken vertically for 60 minutes at 4°C. Subsequently, the mixtures were centrifuged (10,000 × g for 10 min) to isolate the supernatant. A 96-well plate was coated overnight at 4°C with specific capture antibodies, after which any nonspecific interactions were inhibited using a 1% bovine serum albumin (BSA) blocking solution. Standards, quality controls, and GCF eluates (100 μL / well) were dispensed into designated wells. Following incubation and washing, the samples were treated with biotin-conjugated detection antibodies, followed by streptavidin-HRP. After additional washes, the enzymatic reaction was initiated by adding TMB substrate (Solarbio, T8170), and color development proceeded under dark conditions. The reaction was stopped, and optical density (OD) values were measured at 450 nm to determine concentrations. Furthermore, serum interleukin (IL)-1β and IL-6 levels were also assessed via ELISA, as described above.

High-sensitivity C-reactive protein (hs-CRP) was detected by automatic blood cell analyzer (Mindray, BC5800). Preheat the machine, check the reagent allowance and the state of the reaction cup, and perform the liquid circuit flushing. Calibrate the instrument with the matching calibrator (according to the manufacturer’s instructions) before the daily test. After the calibration is passed, the sample can be tested. Two to three levels of internal quality control samples were tested to confirm that the results were within the allowable range (±2SD). The processed sample is put into the autosampler, and the instrument completes automatically: sample adding, reaction, detection, and the instrument automatically outputs the results. Quality control: stable quality control materials of the same type (serum/plasma) as the test samples were used to cover three concentration levels: low (close to the Cut-off value), medium (mean value of reference range) and high (pathological value). The quality control should be checked before daily testing, and the quality control should be reverified after changing reagents, calibrators or instrument maintenance. Precision (within-run CV ≤ 5%, between-run CV ≤ 8%), accuracy (recovery 95% – 105%), linear range (r ≥ 0.99, covering 0.1–100 mg/L), detection limit (≤ 0.1 mg/L).

For T lymphocyte subset profiling, 100 μL of whole blood was combined with a premixed antibody cocktail [CD3-FITC (Clone UCHT1, BioLegend) / CD4-PE (Clone RPA-T4, BD Biosciences) / CD8-APC (Clone SK1, eBioscience) in a flow tube and incubated for 15 minutes in darkness. Subsequently, red blood cells were lysed using a lysis buffer, and the samples were washed with PBS before analyzing T lymphocyte subsets (CD3^+^, CD4^+^, CD8^+^) by flow cytometry. 

### Statistical analysis

Data analysis was conducted using SPSS 25.0. Categorical variables were expressed as [n(%)] and compared using the chi-square test (when P > 0.05, it means normal distribution data, otherwise it means non-normal distribution data). Continuous variables, confirmed to follow a normal distribution via the Shapiro-Wilk test, were presented as (mean ± SD; χ̄±s) and analyzed with independent sample t-tests. Pearson correlation analysis examined relationships between variables, and Bonferroni correction (α=0.025). While receiver operating characteristic (ROC) curves assessed diagnostic performance. Statistical significance was set at P < 0.05.

## Results

### Observation of IRF4 and FGF23 status in DH

**Figure 1:**
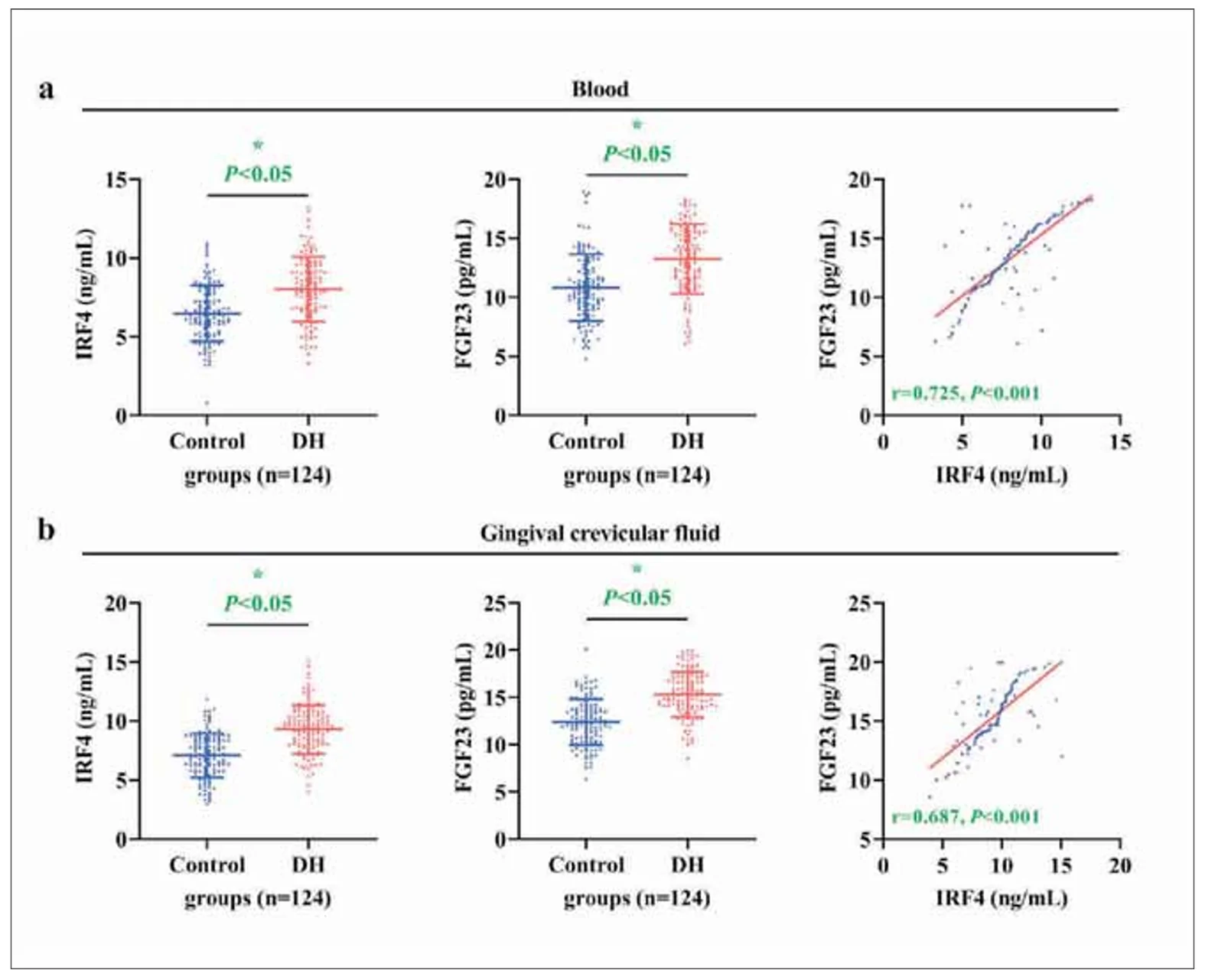
IRF4 and FGF23 expression in DH. a) Comparison and correlation of IRF4 and FGF23 in peripheral blood between DH patients and controls. b) Comparison and correlationof IRF4 and FGF23 in GCF between DH patients and controls. *P < 0.05.

The detection identified significantly upregulated IRF4 and FGF23 expression in DH patients, with both biomarkers showing elevated concentrations in GCF and blood samples compared to controls (P < 0.05). Additionally, these factors displayed a statistically significant co-expression relationship in DH patients (P < 0.05; Figure 1).

### Diagnostic performance of IRF4 and FGF23 in DH detection

**Figure 2:**
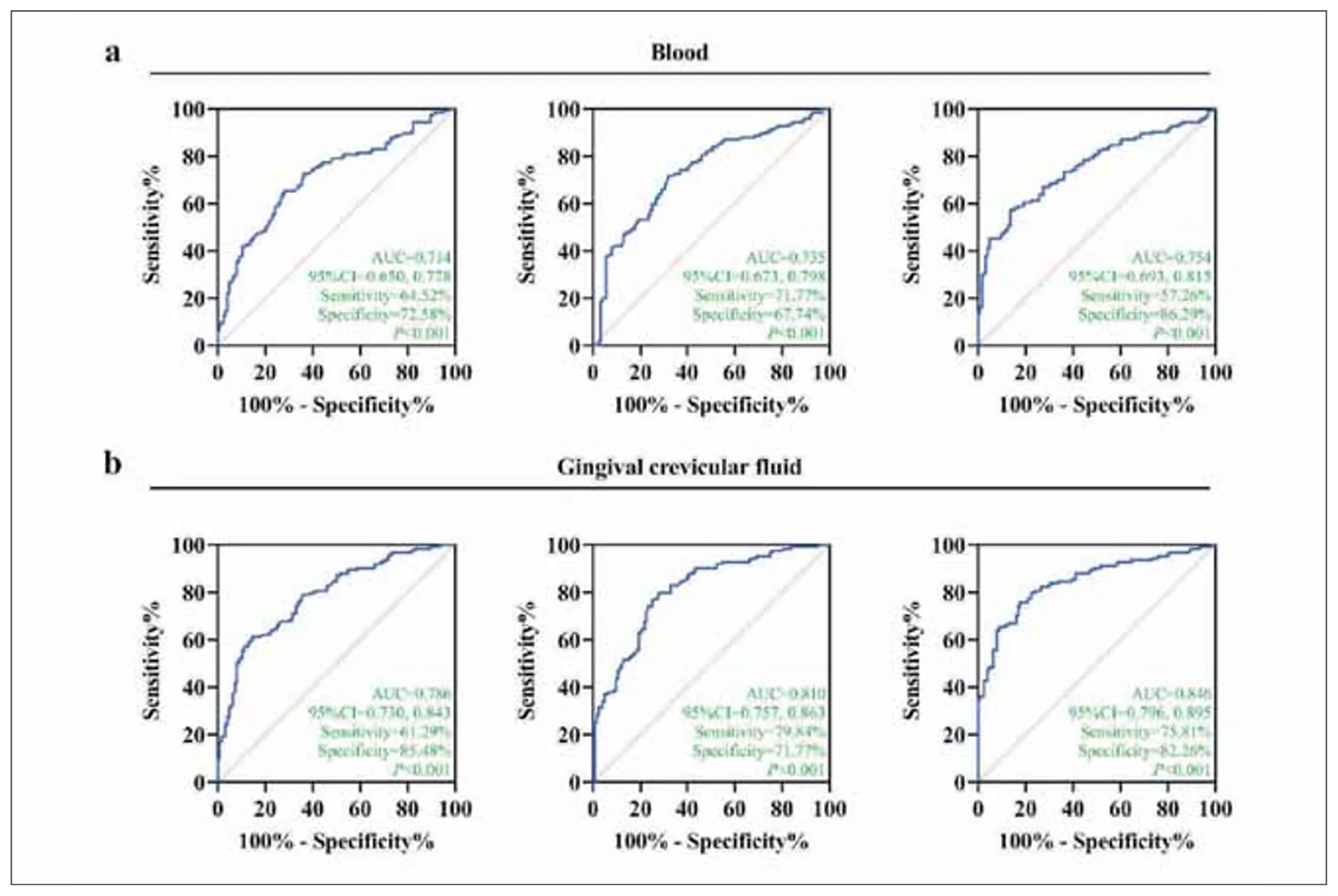
Diagnostic effect of IRF4,FGF23 on DH. a) Diagnostic efficacy of IRF4 and FGF23 in peripheral blood for DH. b) Diagnostic efficacy of IRF4, FGF23 in GCF for DH.

ROC curve analysis revealed that serum IRF4 (cut-off: >7.34 ng/mL) and FGF23 (cut-off: >11.53 pg/mL) exhibited diagnostic sensitivities of 64.63% and 75.16%, respectively, with specificities of 64.52% and 71.77%. Using Logistic regression analysis, we established the formula for the combined detection of IRF4 and FGF23 in serum samples: -4.601+0.293×IRF4+0.208×FGF23 (Table 2). When the two were combined (cut-off: >0.57), the diagnostic sensitivity for DH was reduced (57.26%), but the specificity was increased to 86.29% (P < 0.05). Although the AUC of combined detection was higher than that of single detection, the improvement in diagnostic efficiency was not significant. Notably, IRF4 (cut-off: >8.71 ng/mL) and FGF23 (cut-off: >13.59 pg/mL) levels in GCF demonstrated superior diagnostic performance compared to serum (AUC: 0.786 and 0.810, respectively). Similarly, a joint detection formula for GCF samples was established based on the results of regression analysis: -8.587+0.403×IRF4+0.385×FGF23 (Table 2). The combined use of GCF-based IRF4 and FGF23 (cut-off: >0.52) further enhanced detection, yielding a sensitivity of 75.81% and specificity of 82.26% (AUC=0.846, P < 0.05) (Figure 2).

**Table 2:** Analysis of the effects of IRF4 and FGF23 on DH in blood samples and GCF samples (Logistic regression analysis).

Samples	Indicators	B	SE	Wals	OR (95%CI)	P
Serum	IRF4 (ng/mL)	0.293	0.084	12.210	1.340 (1.137, 1.579)	<0.001
	FGF23 (pg/mL)	0.208	0.054	14.953	1.231 (1.108, 1.368)	<0.001
	Constant	-4.601	0.744	38.238	-	-
GCF	IRF4 (ng/mL)	0.403	0.093	18.819	1.496 (1.247, 1.795)	<0.001
	FGF23 (pg/mL)	0.385	0.075	26.433	1.470 (1.269, 1.703)	<0.001
	Constant	-8.587	1.135	57.236	Not reported	Not reported

### Association of IRF4 and FGF23 with inflammatory markers

**Figure 3:**
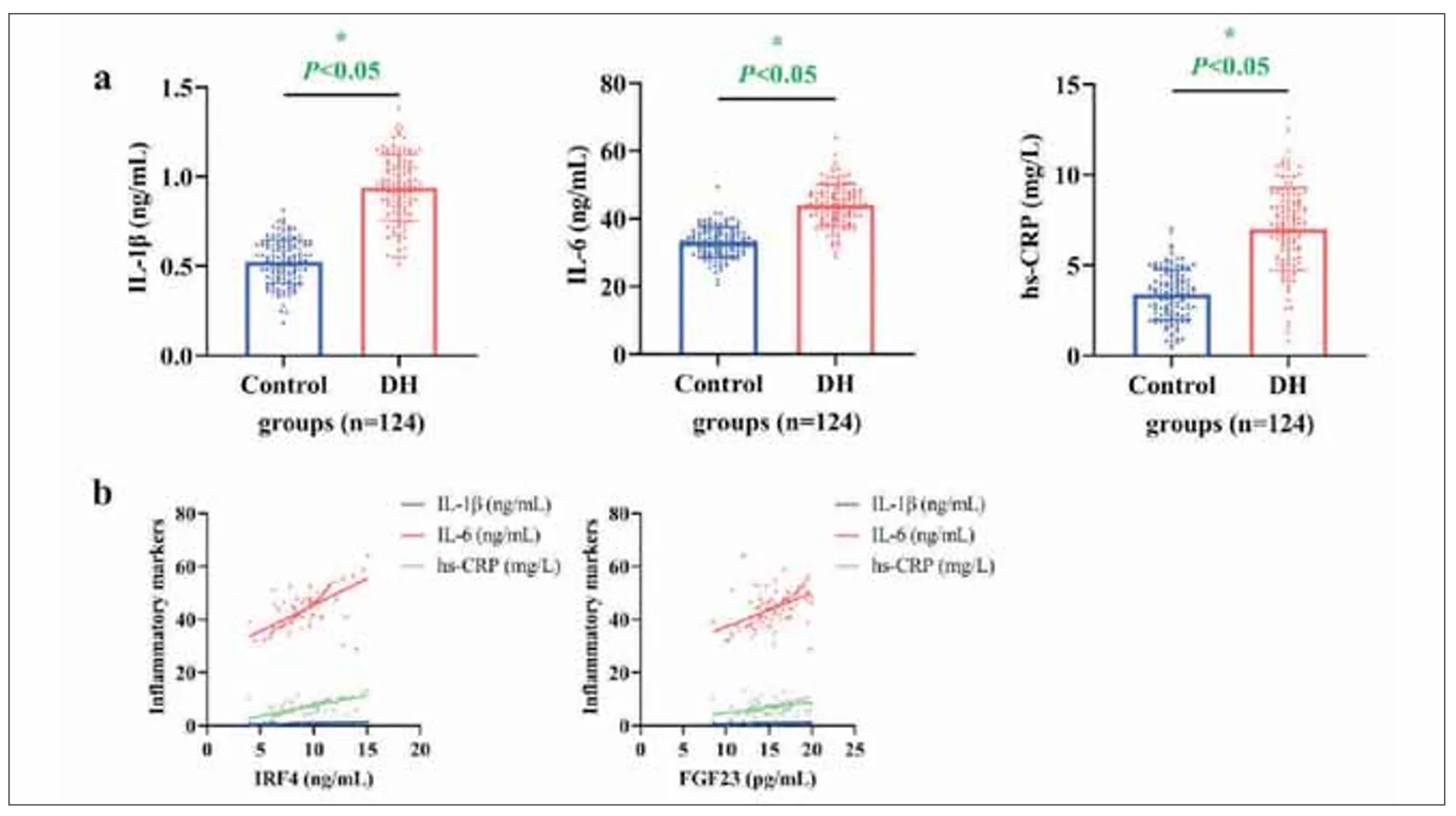
Relationship between IRF4, FGF23 and inflammatory factors in DH patients. a) Comparison of IL-1β, IL-6, and hs-CRP between DH patients and controls. b) Correlation of IRF4, FGF23 and IL-1β, IL-6, hs-CRP inDH patients. *P < 0.05.

Regarding inflammation, the DH group demonstrated increased inflammatory markers (IL-1β: 0.94±0.18 ng/mL, IL-6: 44.04±6.06 ng/mL, hs-CRP: 7.00±2.30 mg/L) relative to controls (P < 0.05). Positive correlations were observed betweenGCF IRF4/FGF23 concentrations and these inflammatory mediators (P < 0.05), indicating their potential role in exacerbating inflammatory processes (Figure 3).

### Relationship between IRF4, FGF23 and T lymphocyte subsets

**Figure 4:**
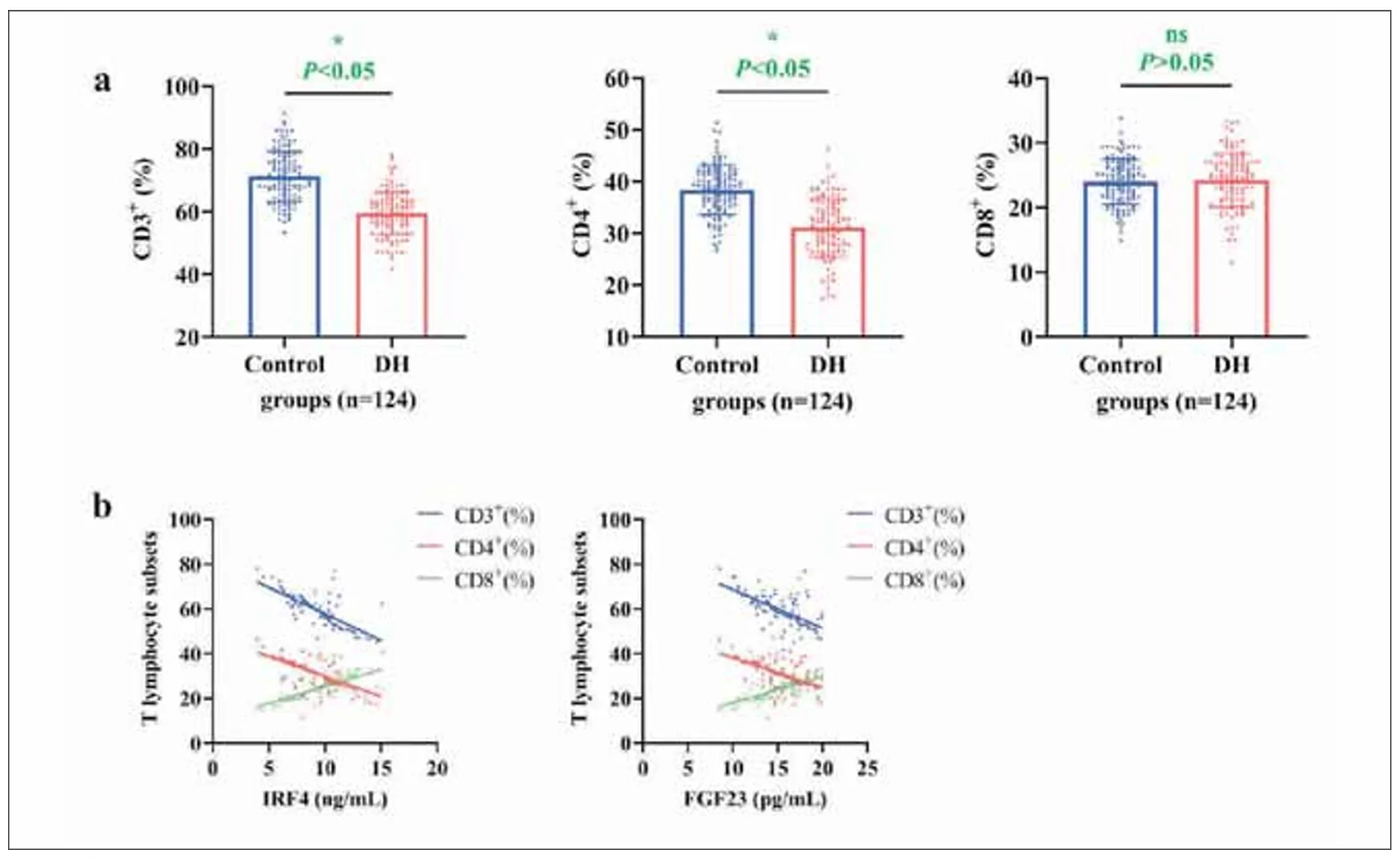
Relationship between IRF4, FGF23 and T lymphocytes in DH patients. a) Comparison of CD3^+^, CD4^+^, and CD8^+^ between DH patients and controls. b) correlation between IRF4, FGF23 and CD3^+^, CD4^+^, CD8^+^ in DH patients. *P < 0.05, nsP > 0.05.

Finally, the comparison of T lymphocyte subsets revealed no significant difference in CD8^+^ levels between the groups (P > 0.05). However, CD3^+^ and CD4^+^ counts were significantly reduced in the DH group compared to controls (P < 0.05). Notably, IRF4 and FGF23 levels in GCF exhibited a negative correlation with CD3^+^ and CD4^+^, and positively correlated with CD8^+^ (P < 0.05), implying that elevated IRF4 and FGF23 may contribute to T lymphocyte subset dysregulation (Figure 4).

## Discussion

This research is the first to demonstrate a strong association between GCF IRF4 and FGF23 levels and DH severity, as well as the enhanced diagnostic accuracy of the IRF4-FGF23 combined detection versus their individual measurements.

Regarding molecular mechanisms, IRF4 is a core driver of immune microenvironment remodeling. Functioning as an immunoregulatory hub molecule, IRF4 plays a dual role in the pathological progression of DH. First, it influences inflammatory responses by modulating the Th17/Treg balance [13], as evidenced by the significant positive correlation between GCF IRF4 levels and the pro-inflammatory cytokines IL-1β and IL-6 in DH patients. This finding aligns with previous animal studies showing impaired Th17 cell differentiation in IRF4 knockout mice [14]. Second, IRF4 enhances the release of inflammatory mediators by activating the NF-κB axis [15], which explains the observed positive association between IRF4 and hs-CRP levels in this study. FGF23, primarily functions through suppressing phosphate reabsorption in renal tubules, yet its profound influence on bone metabolism holds greater clinical relevance [16]. This study reveals the relationship between IRF4, FGF23 and DH, and we speculate that the mechanism may be related to the following pathways: (1) Impaired Dentin Mineralization: By suppressing alkaline phosphatase (ALP) activity, FGF23 disrupts hydroxyapatite crystal formation [17], a finding aligned with our observed correlation between elevated FGF23 and increased dentinal tubule exposure. (2) Osteoclast Activation: Through the RANKL/OPG axis, FGF23 potentiates osteoclast function [18]. (3) Dental Pulp Microenvironment Dysregulation: Dental pulp stem cells express FGF23 receptors [19], and elevated FGF23 levels may hinder pulp repair processes. We were also the first to identify a strong positive association between IRF4 and FGF23 in GCF, indicating a potential collaborative role in disease progression. One possible explanation is that inflammation disrupts mineralization homeostasis, where IRF4-driven inflammatory conditions could upregulate FGF23 via ROS-dependent MAPK signaling. Alternatively, FGF23-induced dentinal tubule exposure might heighten TRPV1 channel sensitivity through hydrodynamic mechanisms [20], establishing a regenerative feedback loop of »inflammation-hypersensitivity-recurrent inflammation« that exacerbates inflammatory responses due to impaired mineralization. Additionally, the observed reduction in CD3^+^ and CD4^+^ T cells in DH patients correlated with elevated IRF4/FGF23 levels, suggesting their immunomodulatory functions. It has also been mentioned in previous studies that IRF4 may indirectly inhibit CD4^+^ T cell differentiation through STAT3 pathway [21], which explains the results in this study. These insights highlight IRF4 and FGF23 as promising therapeutic targets for DH management.

Our clinical observations further indicated that measuring IRF4 and FGF23 in GCF provided a more accurate diagnosis of DH than serum-based testing. When both biomarkers were combined for assessment, they achieved an impressive AUC of 0.846, underscoring their clinical utility. This enhanced diagnostic capability likely arises from GCF’s distinct biochemical characteristics as a site-specific inflammatory exudate. Three key mechanisms may explain this advantage: First, the localized pressure differential near exposed dentin promotes selective migration of inflammatory molecules into GCF [22], leading to targeted enrichment. Second, GCF possesses a unique proteolytic barrier, where matrix metalloproteinases (MMPs) preferentially degrade blood-derived proteins [23], maintaining a cleaner local biomarker profile. Third, with a rapid renewal rate (4–6 hours), GCF more effectively and sensitively captures acute inflammatory shifts compared to slower-turnover biofluids. However, further validation is needed, as this study did not include longitudinal tracking of IRF4 and FGF23. According to these findings, GCF-based detection of IRF4 and FGF23 could potentially serve as biomarkers for clinical diagnosis or prognostic assessment of DH, instead of cold stimulation test. Future research may explore therapeutic interventions targeting the IRF4/FGF23 pathway, offering novel treatment approaches for DH patients. These insights hold significant promise for advancing the diagnosis and management of DH in clinical settings.

This study focused on orthodontic patients aged 18-30, excluding high-risk groups for DH, such as middle-aged and elderly individuals or those with caries or periodontal diseases. At the same time, due to the lack of data from middle-aged and elderly DH patients, the extrapolation of the results should be cautious. This limited scope may impact the generalizability of the findings. Additionally, while ELISA offers convenience and rapid detection capability, supplementary validation employing diverse analytical approaches would strengthen the reliability of key pathway proteins. Moreover, in vitro studies – such as experiments involving dentin cell lines – were not conducted to assess the direct role of IRF4/FGF23. Future research should address these limitations, and monitor the dynamic changes of biomarkers after desensitization treatment (e.g., 1/3/6 months).

## Conclusion

The detection of IRF4 and FGF23 in GCF could be used to diagnose DH more accurately than serum samples. Meanwhile, IRF4 and FGF23 are closely related to inflammatory factors and T lymphocyte subsets in DH patients. The results contribute to elucidating DH’s pathogenesis while offering valuable experimental evidence for future targeted diagnostics and therapies.

## Dodatak

### Consent to publish

All authors gave final approval of the version to be published.

### Availability of data and materials

The data that support the findings of this study are available from the corresponding author upon reasonable request.

### Acknowledgements

Not applicable.

### Conflict of interest statement

All the authors declare that they have no conflict of interest in this work.
